# Supplementary material for: The Single-Stranded DNA-Binding Gene Whirly (Why1) with a Strong Pathogen-Induced Promoter from Vitis pseudoreticulata Enhances Resistance to Phytophthora capsici
Source: Int J Mol Sci. 2022 Jul 21;23(14):8052. doi: 10.3390/ijms23148052 (PMC9315732; doi:10.3390/ijms23148052)
Supplement: Supplementary file 1 [file ijms-23-08052-s001.zip › ijms-1785706-supplementary.pdf]

International Journal of *Molecular Sciences*

MDPI

Supplementary Material

The Single-Stranded DNA-Binding Gene *Whirly* (*Why1*) with a Strong Pathogen Induced Promoter from *Vitis pseudoreticulata* Enhances Resistance to *Phytophthora capsici*

Chengchun Lai [1], Qiuxia Que [1], Ruo Pan [1], Qi Wang [1], Huiying Gao [1], Xuefang Guan [1], Jianmei Che [2,*] and Gongti Lai [1,*]

1 Institute of Agricultural Engineering Technology, Fujian Academy of Agricultural Sciences, Fuzhou 350003, China; lccisland@163.com (C.L.); queqiuxia2020@163.com (Q.Q.); rrpanpan@163.com (R.P.); nkywq@163.com (Q.W.); faas14061407@163.com (H.G.); guan-619@163.com (X.G.)

2 Institute of Agricultural Bio-Resources Research, Fujian Academy of Agricultural Sciences, Fuzhou 350003, China

* Correspondence: chejianmei@faas.cn (J.C.); laigongti@faas.cn (G.L.)

Supplementary Table

Table S1: Sequence information *of Why1* and promoter

Genes/ promoters	GenBank ID	Sequence length/bp	ORF length/bp	Number of exon/intron	Amino acid number	Molecular weight/kD	Theoretical pI	Hydro- pathicity	Subcellular localization
VvWhy1	MN395403	862	807	7/6	268	68.33	5.1	0.762	Chloroplast
VpWhy1	MN395402	862	807	7/6	268	68.28	5.1	0.755	Chloroplast
VvWhy1 promoter	MN397251	1127							
VpWhy1 promoter	MN397250	1136							

Table S2: Primer sequence

Primer name	Primer sequence	Vector or purpose
Why1-F	GGTTAAGAAGAATAGAGGCGGAAG	Gene cloning of *VvWhy1* and
Why1-R	CAGTCCATGCTTTAGAAGTACCAG	*VpWhy1*
p-F	TGAGTTATGACTTTATTGTTTCTCCTT	Promoter cloning of *pVv* and
p-R	GATGCATTCTTCCGCCTCTATTCTTCT	*pVp*
Why1-gfp-F	CGGGATCCATGCATCACCTGCACCTGCTTTC	pBI121-*Why1-GFP*
Why1-gfp-R	GGGGTACCCCTCTGCTCCATTCAAAGTCCCCAG	
p-GUS-F	CCCAAGCTTTGAGTTATGACTTTATTGTTTCTC	pBI121-*pWhy1::GUS*
p-GUS-R	TCCCCCGGGTCTTCCGCCTCTATTCTTCTTAAC	
p-Why1-gfp-F	ATGCCTGCAGGTGAGTTATGACTTTATTGTTTCTC	pBI121-*pWhy1::Why1-GFP*
p-Why1-gfp-R	CGGGATCCTCTTCCGCCTCTATTCTTCTTAAC	
Why1-q-F	AGCAGAACATCACTCGCA	q-RT-PCR
Why1-q-F	TTGGGCTCTACCGTAAGA	
NbPRl-q-F	CCGTTGAGATGTGGGTCAAT	
NbPRl-q-R	CGCCAAACCACCTGAGTATAG	

NbPR2-q-F	CAACCCGCCCAAAGATAGTA
NbPR2-q-R	TGGCTAAGAGTGGAAGGTTATG
NbPR4-q-F	GGATGATGTTGACAGCAGAGA
NbPR4-q-R	GTAGGACACGAGGTAGGTATCA
NbPR5-q-F	GCTCGATTACGTCTTGTCTCTC
NbPR5-q-R	CTCTAGCATGGTGGATTGACTT
NbPR10-q-F	GAAGAAGAACACAATGAAGGCA
NbPR10-q-R	CAGTAGGATTGGCAAGAAGGTA
V-EF1α-F	GAACTGGGTGCTTGATAGGC
V-EF1α-R	ACCAAAATATCCGGAGTAAAAGA
Nb-EF1α-F	AGAGGCCCTCAGACAAAC
Nb-EF1α-R	TAGGTCCAAAGGTCACAA

Red letters indicate restriction enzyme sites, GGATCC:*Bam*HI; GGTACC: *Kpn*I; AAGCTT: *Hin*dIII; CCCGGG: *Sma*I; *Sbf*I:CCTGCAGG.

Why1-gfp-F and *Why1-gfp-R* were used to construct pBI121-*VvWhy1-GFP* and pBI121-*VpWhy1-GFP* for subcellular localization verification.

p-GUS-F and *p-GUS-R* were used to construct pBI121-*pVv::GUS* and pBI121-*pVp::GUS* for promoter GUS activity assay.

Based on the constructs of pBI121-*VvWhy1-GFP* and pBI121-*VpWhy1-GFP*, *p-Why1-gfp-F* and *p-Why1-gfp-R* were used to construct pBI121-*pVv::VvWhy1-GFP*, pBI121-*pVv::VpWhy1-GFP*, pBI121-*pVp::VvWhy1-GFP* and pBI121-*pVp::VpWhy1-GFP* for *Ph. Capsici* resistance experiments in *N. benthamiana*.

Supplementary Sequence

Sequence S1: *VvWhy1*

>*VvWhy1* ORF 26-832 bp

GGTTAAGAAGAATAGAGGCGGAAGAATGCATCACCTGCACCTGCTTTCTTCCTCATTCACCATCCAAAACCC
TAGATTATGCCCTAATCACTCCCTTTCCTCGCTCCACTCCTCCTCACCCTTGAGCTTCACTTCACGAACTCCTCT
TCTGCTTTCCACAACCAGGCTCTTCCGTAAAAAGCGCTCATTGCAATGTCGCCAGTCGGATTACTTTCAACAG
CAGAACATCACTCGCAGACAACCTCCAAATGACTCTTCTTTTGGAGGAGCTTTGCAGCCTAGGGTTTTTGTTG
GTCATTCGATATACAAAGGGAAAGCTGCTCTTACGGTAGAGCCCAAAGCCCCGGAGTTTACACCTTTAGATT
CAGGGGCATTTAAAGTGTCCAAAGAAGGTTTTGTGCTGCTCCAGTTTGCTCCTGCTGCAGGTGTTCGACAATA
TGATTGGGGCAGAAAGCAGGTGTTCTCATTATCTGTGACTGAAATTGGAAGTCTTATTAGCCTTGGTGCAAGA
GAGTCATGCGAATTTTTTCATGATCCTTTTAAAGGAAGAAGTGAGGAAGGTAAGGTCCGGAAGGTGTTGAAG
GTAGAGCCGCTCCCAGATGGCTCTGGTCATTTCTTCAATCTCAGTGTTCAAAACAAGCTTTTGAATATGGATG
AGAACATTTATATCCCTGTCACCAGAGCAGAATTTGCTGTGCTCATCTCAGCTTTTAATTTCATTGTGCCATAT
CTTTTAGGCTGGCATGCCTATGCGAACTCCATCAAGCCAGATGATACAAGTCGTGTGAATAATGCTAATCCGA
GATCTGGGGACTTTGAATGGAGCAGATAGTAAACACTGGTACTTCTAAAGCATGGACTG

Sequence S2: *VpWhy1*

>*VpWhy1* ORF 26-832 bp

GGTTAAGAAGAATAGAGGCGGAAGAATGCATCACCTGCACCTGGTTTCTTCCTCATTCACCATCCAAAACCC
TAGATTATGCCCTAATCACTCCCTTTCCTCGCTTCACTCCTCCTCACCCTTGAGCTTCACTTCACGAACTCCTCT
TCTGCTTTCCACAACCAGGCACTTCCTTAAAAAGCGCTCATTGCAATGTCGCCAGTCGGATTACTTTCAACAG

CAGAACATCACTCGCAGACAACCTCCAAATGACTCTTCTTTGGAGGAGCTTTGCAGCCTAGGGTTTTGTTG
GTCATTCGATATACAAAGGGAAAGCTGCTCTTACGGTAGAGCCCAAAGCCCCGGAGTTTACGCCTTTAGATTC
AGGGGCATTTAAAGTGTCCAAAGAAGGTTTTGTGCTGCTCCAGTTTGCTCCTGCTGCAGGTGTTCGACAATAT
GATTGGGGCAGAAAGCAGGTGTTCTCATTATCTGTGACTGAAATTGGAAGTCTTATTAGCCTTGGTGCAAGAG
AGTCATGCGAATTTTTTCATGATCCTTTTAAAGGAAGAAGTGAGGAAGGTAAGGTCCGGAAGGTGTTGAAGG
TAGAGCCGCTCCCAGATGGCTCTGGTCATTTCTTCAATCTCAGTGTTCAAAACAAGCTTTTGAATATGGATGA
GAACATTTATATCCCTGTCACCAGAGCAGAATTTGCTGTGCTCATCTCAGCTTTTAATTTCATTGTGCCATATCT
TTTAGGCTGGCATGCCTATGCGAACTCCATCAAGCCAGATGATACAAGTCGTGTGAATAATGCTAATCCGAG
ATCTGGGGACTTTGAATGGAGCAGATAGTAAACACTGGTACTTCTAAAGCATGGACTG

Sequence S3: *VvWhy1* promoter

>*VvWhy1* promoter (pVv) 1127 bp
TGAGTTATGACTTTATTGTTTCTCCTTTATACACAATTATAGTTTTGAATTTAGATAAATAGATTATAAATTAA
GTAAGAAATAATTAATAATTTAAATTCTTTAATAATTTTAAATAAAATTTAAGAATAATTTAGAATAAAAAAA
CCAATTTAATTAATTGTAAATTAAATTAAAAACATTTTAAAATATTATATTGTAATTCAATACTAAATGTGTAC
ACGAAATAAAAAACTTGACTTATTTACGTATTAAAAAAGTTAAAATTTATTATTTATTCATTTTAAATAAAAT
ATTTCTAAAACTTATTATTCTAAATTGTTATTATTCATTTAAGAAAAAAAAATTACGAAGATGTTAATATCCTAT
GGTTTTAATATATACTAAAATAATATTTTTTTAATAAAAAGATATAATTTATGATTTTATTATAATATTATTATT
AATGTTTTATTAAAAACTATATATTTTTTAATTTATTTGTAATTACAAAACTATTTTTATTGTTAATAAGACTTG
AATTAAATTTAATTAATATTATATAAATTGAATTAGTAATGTTTTTATAAAATAAAAAAAAAATCAATTTTAG
AAACAGTTTATCCAAGAAAATCAAATCATTTTATACAAACAAGTTTTTGTTTTTTATTTTGAAAAAAGTTTTTA
AAAAACAATTAGTCAAACACTCTTATATTTATAAAATACAAGAAAACTGTTTTATATTCTTTAATTTAAAAAC
AATTTTTTAAAAACAAATTTAAGAAAATATGACCAAATAGATCCTAATTTTTTTGGAAATAATTCTTAAAAAT
AATTTTTTATTTTCAATAATATATTTTTTTTGTCCCAACTTAATTTTTATGAAAATATCTCATATAGTGTAAATTT
CGAAAAATAACATATTCTCAAAACAAATTTTCAAAAAAAATTTGTATTAAAAATTTAAAGGGGCTTGAGATT
TCCTAAGATGCCAACAGACTTTTTATTCTCACGAAATTAACTTTTGTAATATAGTAATAAGAAACGGCAGCGT
GCCTCAGGGCATCGTCAGCAGCCTCATTGCTTCAAAGTTAAAAGCTCTATCTTCCGAAGCGAAATTGGGGGTT
AAGAAGAATAGAGGCGGAAGAATGCATC

Sequence S4: *VpWhy1* promoter

>*VpWhy1* promoter (pVp) 1136 bp
TGAGTTATGACTTTATTGTTTCTCCTTTATACACAATTATAGTTTTGAATTTAGATAAATAGATTATAAATTAA
GTAAGAAATAATTAATAATTTAAATTCTTTAATAATTTTTAAAAATTTTTAAGAATAATTTAGAATTCAAAAA
AACCAATTTAATTAATTGTAAATTAAATTAAAAACATTTTAAAATATTATATTGTAATTCAATACTAAATGTGT
ACACGATATAAAAAACTTGACTTATTTACATATTAAAAAAGTTAAAATTTATTTTTTATTCATTTTAAATAAAC
TATTTCTAAAAAATTATTATTCTAAATTGTTATTATTCATTTAAGAAAAAAAAATTGAAGATGTTAATATCCTTA
TGGTTTTAATATATAATAAAATAATATTTTTTTTAATAAAAAAATATAATTTATGATTTTATTATAATATTATTA
TTAATGTTTTATTAAAAACTATATATATTTTTAATTTATTTGTAATTAAAAAAACTATCTTTATTGTTAATAAGA
CTTGAATTAAATTTAATTAATATTATATAAATTGAATTAGTAATGGTTTATAAAATCAAAAAAAGAAAAAATC
ATTTTTAGAAACAGTTTATTCAAGAAAATCAAATCATTTTATACAAACAAGTTTTTTGTTTTTTATTTTGAAATT
TTTTTTAAAAAACAATTCGTCAAACACTCTTATATTTATAAAACACAAGAAAACTGTTTTATATTTTTTAATTT
AAAAACAATTTTTTAAAAACAAATTTAAAAAGATATGACCAAATAGACCCTAATTTTTTTGAAAATAATTCTT

AAAAATAATTTTTTATTTTCAATAATATTTTTTTTTTGTCCCAACTTAATTTTTATGAAAATATCTCATATAGTGT
AAATTTCAAAAAATAACATATTCTCAAAACGAATTTTCAAAAAAAATTTGTATTAAAAGTTTAAAGGGGCTT
GAGATTTCCTAAGATGCCAGCAGACTTTTTATTCTCACGAAATTAACTTTTGTAATTTAGTAATAAGAAACGG
CAGCGTGCCTCAGGGCATCGTCAGCAGCCTCATTGCTTCAAAGTTAAAAGCTCTATCTTCCGAAGCGAAATTG
GGGGTTAAGAAGAATAGAGGCGGAAGAATGCATC
